# When agreement-accepting free-riders are a necessary evil for the evolution of cooperation

**DOI:** 10.1038/s41598-017-02625-z

**Published:** 2017-05-30

**Authors:** Luis A. Martinez-Vaquero, The Anh Han, Luís Moniz Pereira, Tom Lenaerts

**Affiliations:** 1 AI lab, Computer Science Department, Vrije Universiteit Brussel, Pleinlaan 2, 1050 Brussels, Belgium; 2 MLG, Département d’Informatique, Université Libre de Bruxelles, Boulevard du Triomphe CP212, 1050 Brussels, Belgium; 3 School of Computing, Teesside University, Borough Road, Middlesbrough, TS1 3BA UK; 4 NOVA Laboratory for Computer Science and Informatics, Departamento de Informática, Faculdade de Ciências e Tecnologia, Universidade Nova de Lisboa, 2829-516 Caparica, Portugal; 5 Institute of Cognitive Sciences and Technologies, National Research Council of Italy, via San Martino della Battaglia 44, 00185 Rome, Italy

## Abstract

Agreements and commitments have provided a novel mechanism to promote cooperation in social dilemmas in both one-shot and repeated games. Individuals requesting others to commit to cooperate (proposers) incur a cost, while their co-players are not necessarily required to pay any, allowing them to free-ride on the proposal investment cost (acceptors). Although there is a clear complementarity in these behaviours, no dynamic evidence is currently available that proves that they coexist in different forms of commitment creation. Using a stochastic evolutionary model allowing for mixed population states, we identify non-trivial roles of acceptors as well as the importance of intention recognition in commitments. In the one-shot prisoner’s dilemma, alliances between proposers and acceptors are necessary to isolate defectors when proposers do not know the acceptance intentions of the others. However, when the intentions are clear beforehand, the proposers can emerge by themselves. In repeated games with noise, the incapacity of proposers and acceptors to set up alliances makes the emergence of the first harder whenever the latter are present. As a result, acceptors will exploit proposers and take over the population when an apology-forgiveness mechanism with too low apology cost is introduced, and hence reduce the overall cooperation level.

## Introduction

Commitments for enhancing cooperation are widespread in human societies^1–4^ at different scales, ranging from personal relationships to international agreements, and from hunter-gatherer to industrialised societies^2–7^. They offer an alternative pathway for the evolution of cooperation as opposed to punishment and rewards^8–20^, deterring defection and antisocial behaviours from free-riders. Commitments provide a way to clarify intentions or preferences of others prior to an interaction^3, 21, 22^, offering a proactive approach for partner choice^17, 23^ – an important mechanism for promoting evolution of cooperation in one-shot interaction which is usually enacted through the reputation effect^24–27^ or spatial interactions^28–32^. In general, the commitment mechanism does not assume kin relationships, repeated interactions or reputation effects, which are some other major factors responsible for the evolution of cooperation^33–37^.

In general terms, commitments can be defined as agreements to cooperate, with posterior compensations when any of the parties involved defects while the others honour the agreements^38, 39^. Strategies examined in this set-up range from the traditional unconditional cooperative and defecting strategies to strategies that, on the one hand, propose, accept or refuse an agreement and, on the other hand, cooperate honouring the agreement or defect in an attempt to exploit the commitment proposer. Strategies can also be conditional on the existence of a commitment, and moreover, as setting up agreements is costly, on whom is paying the cost. Analytical and numerical approaches have shown that commitment proposing strategies emerge for specific set-up and compensation costs, promoting cooperation both in pairwise and in group interactions, and in one-shot as well as repeated games^7, 38, 40–42^. Behavioural experiments support these predictions^21, 43–45^ and when mistakes can be made (*i*.*e*. playing the action opposite to what was intended) in repeated social dilemmas, commitments, and as a consequence cooperation, benefit from apology-forgiveness mechanisms, requiring that the apology be sincere (*i*.*e*. costly enough) for the procedure to be a positive incentive towards cooperation^41, 46–48^.

As commitment proposing behaviour requires others that accept the proposal, one can also wonder whether acceptors can get away with making no effort (paying no setup cost) to make commitment possible. In other words, are they needed to ensure the survival of proposers or can proposers manage among themselves? Moreover, the commitment cost can be spent before or after one knows the acceptance intention of co-players. It is unknown how this difference influences the outcome of the evolutionary dynamics: spending an effort before knowing may be detrimental as it can be exploited by others. Answers to these questions would provide more in-depth understanding of the role that commitment proposing behaviour plays in producing pro-social behaviours. In order to investigate these questions, the mathematical and numerical analysis of the evolution of commitment behaviour has to go beyond the evolutionary models that have been used so far: none of the theoretical work on commitments has examined whether strategies are required to coexist in so-called mixed states, combining for example proposers and free-riding acceptors. The focus has been on Markovian processes where, given enough time, a monomorphic population state is always reached. Yet these absorbing states can be virtually unreachable since the necessary time to actually reach them grows exponentially with the number of individuals in the case of metastable states^37^. Including mixed states would therefore provide a clarification of the expected outcomes of the evolutionary dynamics, ensuring a more correct view of the system dynamics under investigation. Some strategies may be more successful when co-occurring with others, a dynamic outcome that remains invisible if only monomorphic states are considered.

We use herein the ecosystem assembly technique, originally proposed by Capitan and colleagues^49^ to study the ecological dynamics among different communities of species. Their model offers an exact description of the set of microstates and the dynamical pathways of the assembly process, and it does not have the limitation of computing only the averages over a small set of realisations as in standard assembly models. This approach was used afterwards to study the dynamics of one-memory step strategies^37^ in repeated Prisoner’s Dilemma-like games^50^, showing that mixed states play an important role in the dynamics of the system, either arising as the most frequent states in equilibrium or altering the stationary distribution of the pure states.

Using the ecosystem assembly technique we study here which mixed states emerge within the context of commitment strategies both in one-shot and repeated games and analyse their influence on the dynamics of the system in order to answer the previously identified questions. We study mainly two possible commitment models: one where proposers pay the cost of commitment when they already know the acceptance intention of the opponent and another where they spent the cost before knowing. Our results reveal that the outcome differs between these two models as well as between one-shot and repeated games, making the conclusions non-trivial.

## Results

### Variations in commitment models

The commitment model consists of two stages: In the first stage players can set up commitments^38, 41^. In the second stage they play the Donation game^37^ – an instance of the prisoner’s dilemma.

We analyse separately different commitment mechanisms that can be set up during the first stage:

- Regular commitments (RC): a commitment is set up when at least one of the players is a proposer. The proposer pays a set-up cost $\epsilon$ (just half if both players are proposers) only if the proposal is accepted by the other player. Once having been set up (*i*.*e*. second stage), any player who defects while her co-other cooperates has to pay a compensation cost *δ* to the other, and the commitment is broken^38^.
- Prior commitments (PC): similar to RC, but the commitment set-up cost is paid in advance before knowing whether both players will commit. This means that proposers always have to pay $\epsilon$ even when the commitment is not finally set up because the other player is a non-committer.

Note that RC captures the commitment scenarios wherein players know a priori if their proposals will be accepted so that they only arrange them when needed. For instance, they can communicate with their potential co-players to check whether they are willing to commit before actually paying the cost of arranging the commitment itself (e.g. paying a lawyer), provided the cost of communication itself is negligible. However, in the real world this is not always true. Investments in the form of money, time, communication or even emotions by the proposers are in many cases already made before knowing if any other individual will agree to participate. The PC case models such scenarios. In the following we will see that this difference plays a significant role for the success of a commitment system. Modelling them both allows us not only to understand various realistic commitment scenarios but also to show that it is crucial to support the easy making of a commitment deal.

We have also analysed a third mechanism –deposit-refund–, but since the results obtained by this mechanism are similar to those arising from RC, we leave its analysis to the Supplemental Material.

In the second stage, players can cooperate (C) or defect (D) and they acquire payoffs according to the following parametrised payoff matrix of the Donation game^37^:


1


where *b* > *c* > 0, with *b* and *c* standing for the benefit and cost of cooperation.

### The strategy space

We study both one-shot and repeated games. In the latter situation we include noise, *i*.*e*. with a given probability α individuals perform the opposite action to what they intended to as defined by their strategy. In repeated games, if individuals play within a commitment agreement and it is broken, they can continue playing. So in this latter case a strategy needs to specify actions both when an agreement is in place and when it is absent, leading to strategy definitions that consist of four parameters: *S*_*i*_ = (*S*_*c*_, *S*_*in*_, *S*_*out*_, *S*_*apo*_)_*i*_, where:

- *S*_*c*_ ∈ {*P*, *A*, *N*} represents whether the strategy is played by a proposer who proposes and accepts commitments (P), an accept or who does not propose commitments but accepts if being proposed (A), or a non-committer that never accepts a commitment proposal nor proposes one (N).
- *S*_*in*_ ∈ {*C*, *D*} indicates the behaviour the player chooses when she is in a commitment. Non-committers do not have any *S*_*in*_ action since they never participate in commitments.
- *S*_*out*_ ∈ {*C*, *D*} represents the action chosen by the player when she is not in a commitment, *i*.*e*. when the commitment is not set up or having been set up was subsequently broken.
- *S*_*apo*_ ∈ {*Ap*, *N*-*Ap*} is introduced only in repeated games as part of an apology-forgiveness mechanism in order to deal with errors in these games^41^. It represents whether the individual apologises after a defection. As in ref. 41, we consider that an individual is automatically forgiven when she apologises. This parameter is only used in repeated games since in one-shot games there cannot be a restorative mechanism by definition.

For example, an individual playing the strategy (*P*, *C*, *D*, *A*) proposes and accepts commitments, tries to cooperate within them, apologizing if she defects (by mistake), but if the commitment is broken or not set up she will defect. Another example is a cheater that plays (*A*, *D*, *D*, *A*); she will accept to be part of a commitment if the other is proposing it but she always defects, apologizing after it in order to exploit the system. Note that in this way we include every possible combination of strategies in our analysis, not assuming which of them are more likely to appear.

### Emergence of mixed states

**Figure 1:**
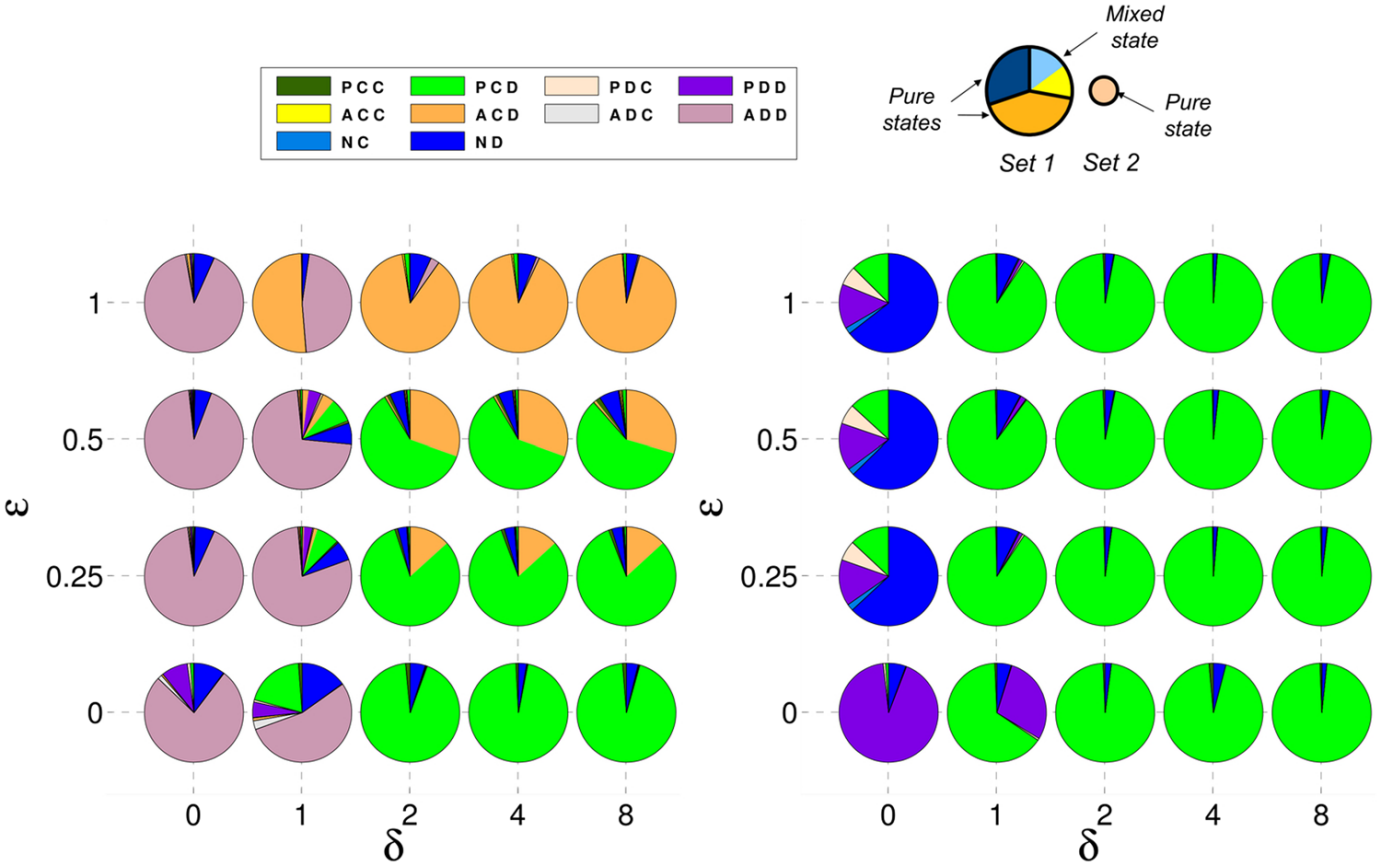
Proposers avoid pure defectors without the need of acceptors in RC games. Composition of the recurrent sets for RC one-shot games. Composition of the recurrent sets for each studied RC type game when all the strategies are included (left) and when acceptors are removed (right). Each pie-chart is associated with a specific ($\epsilon$, *δ*) pair. Each strategy is assigned a different colour. The pieces of the pie-chart separated by thick lines correspond to different nodes of the recurrent set. Their sizes are proportional to their probabilities in the stationary state. If a sector is of a single colour it means that the node corresponds to a pure state; if it is subdivided in smaller sectors with different colours it means that the node corresponds to a mixed state, the different colours representing the coexisting strategies. The sizes of these sub-sectors are proportional to their fraction within the mixed state. On the top-right of this figure, a visual explanation through an illustration in the figure has been included.

**Figure 2:**
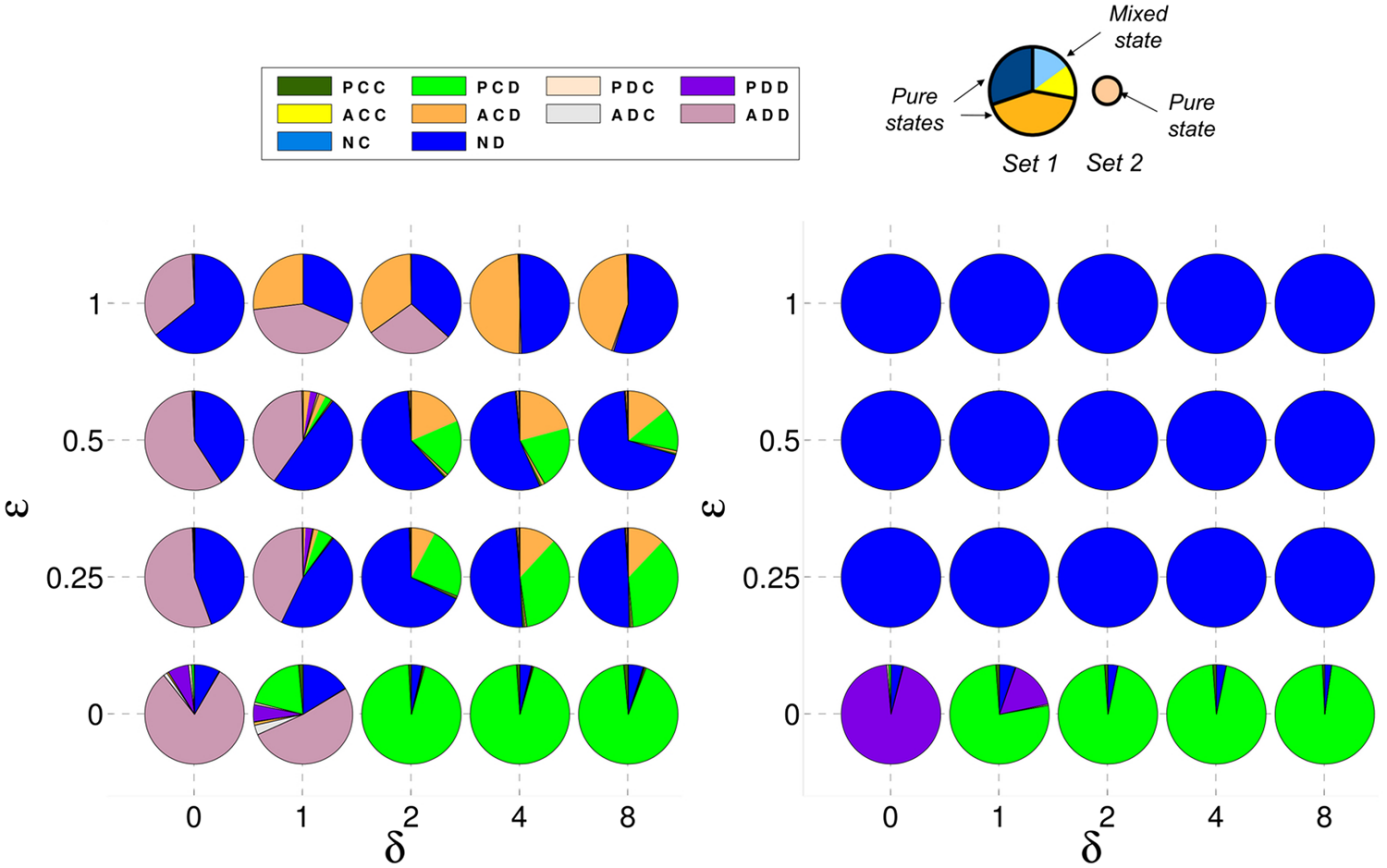
Mixed states between proposers and acceptors are necessary to maintain pure defectors at bay in PC games. Composition of the recurrent sets for PC one-shot games. Same as Fig. 1 but for PC type games.

In Figs 1 and 2 we represent the stationary distribution of strategies for the three types of one-shot games for different values of $\epsilon$ and *δ*. In general, we see that the increase of the compensation δ favours the (*P*, *C*, *D*) strategy. If this value is too low, different defectors –(*N*, *D*), (*A*, *D*, *D*), or (*P*, *D*, *D*)– can take over the population. When E is overly high, acceptors, which do not pay any cost for setting up the commitment, replace proposers and that situation can also favour the appearance of some defectors. These results were also observed in ref. 38.

More importantly, we can see that the main mixed state that appears is formed by (*P*, *C*, *D*) and (*A*, *C*, *D*) strategies, respectively green and orange in the figures. An analytical estimation of the fraction of each strategy in the mixed states can easily be obtained using the replicator dynamics^51^ for all types of games.

The velocity by which the fraction x of one strategy A is growing in the population formed by two strategies *A* and *B* is:

2 $$\dot{x}=x(1-x)({W}_{A}-{W}_{B}),$$

with *W*_*i*_ the average payoff the strategy *i* obtains in that population. It can be computed as *W*_*i*_ = *xW*_*iA*_ + (1 − *x*) *W*_*iB*_, where *W*_*ij*_ is the payoff that i-individuals obtain playing with *j*-individuals. The equilibria are obtained when $\dot{x}=0$ and then only one mixed equilibrium can appear:

3 $$x=\frac{{W}_{AB}-{W}_{BB}}{{W}_{AB}+{W}_{BA}-{W}_{AA}-{W}_{BB}},$$

that corresponds to a fraction of (*P*, *C*, *D*) of $1-\frac{\epsilon /2}{b-c-\epsilon /2}$ in the case of RC games, and $1-\frac{\epsilon }{b-c}$ in PC games. These values match approximately the results obtained through simulations and shown in Figs 1 and 2. Note that we use the replicator dynamics analysis only to illustrate the concrete behavioural dynamics of the main strategies that emerge in the stationary distribution computed through simulations.

### Crucial role of acceptors when agreement costs are made early

**Figure 3:**
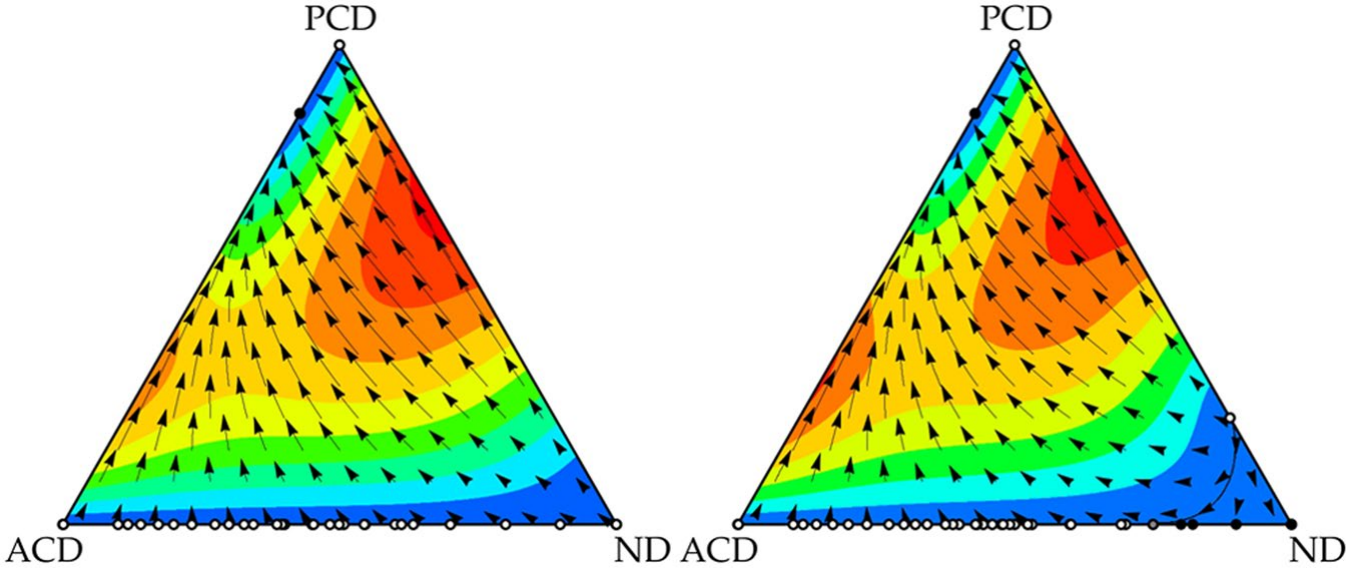
Pure defection is stable in PC games but not in RC games. A mixed state between cooperative proposers and acceptors is stable in both scenarios. Replicator dynamics for a subset of strategies in one-shot games. Replicator dynamics for an infinite population with (*P*, *C*, *D*), (*A*, *C*, *D*), and (*N*, *D*) strategies for RC (left) and PC (right) games. Small filled (empty) circles represent stable (unstable) rest points. We assumed $\epsilon$ = 0.25 and *δ* = 4. Several filled (empty) circles along the ACD-ND axis reveal that the whole interval is stable (unstable) in the axis; the grey filled circle represents the change of stability in the axis for the PC game. Regions where the dynamics lead to a mixed state of (*A*, *C*, *D*) with (*P*, *C*, *D*) or (*N*, *D*) in the PC game are separated by a black curve. Figures obtained with Dynamo^58^.

If we focus on the role of the acceptors, we see that when they are removed from the population in the case of RC (see Fig. 1 right panel), the mixed state formed by (*P*, *C*, *D*) and (*A*, *C*, *D*) is replaced by a (*P*, *C*, *D*) pure state. This result shows that acceptors do not play any role in the restriction of pure defectors in these cases, they just coexist with proposers and reduce slightly the level of cooperation in the system (a mixed state with acceptors is worse for cooperation than a pure state with only cooperative proposers). However, acceptors play a fundamental role in PC games. In this scenario, the mixed state is replaced by pure defectors (see Fig. 2 right panel). In other words, the coexistence of cooperative proposers and acceptors keeps defectors outside the population in the case commitment costs are paid before the agreement is established. In PC games, (*P*, *C*, *D*) obtain less payoff against (*N*, *D*) than in RC games since in the former they have to pay for trying to set up the commitment whereas in the latter both strategies obtain the same payoff. In order to understand better this behaviour, we show in Fig. 3 the dynamics of an infinite population formed by the three main strategies –(*P*, *C*, *D*), (*A*, *C*, *D*), and (*N*, *D*)− according to replicator dynamics for $\epsilon$ = 0.25 and *δ* = 4. One can see that in RC games the mixed state is the only stable equilibrium, but (*N*, *D*) emerges as a new stable point in the PC game. If (*A*, *C*, *D*) is removed from the simplexes, (*P*, *C*, *D*) becomes the only stable point in RC games whereas (*N*, *D*) is also stable in the PC game.

### Absence of mixed states in repeated commitment games

**Figure 4:**
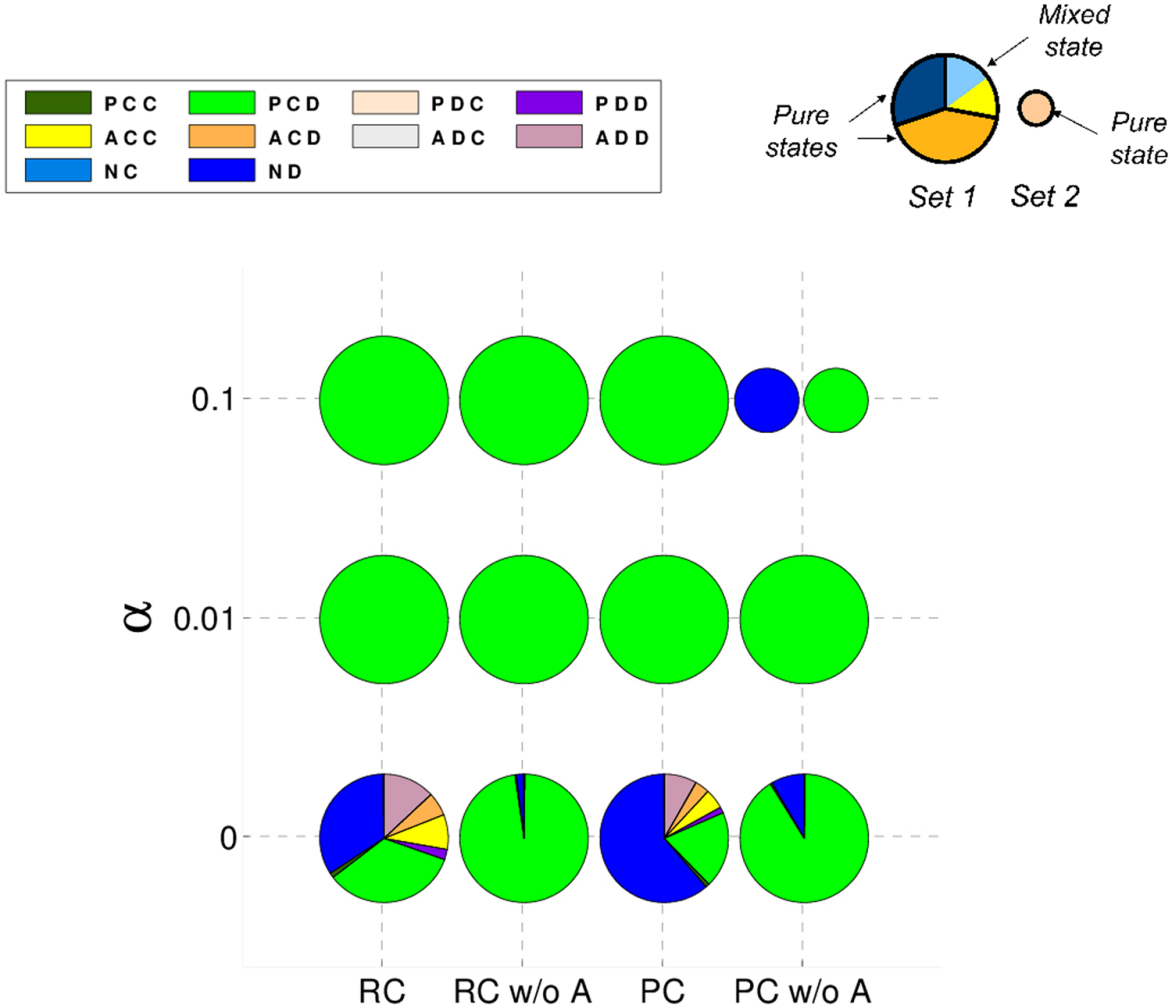
No mixed states are formed in repeated games. Acceptors reduce cooperation in the absence of noise. Composition of the recurrent sets for repeated games without forgiveness. Strategies represented as in Fig. 1 for RC and PC repeated games for different values of α. Different pies for a given game stand for different attractors. We assumed $\epsilon$ = 0.25 and *δ* = 4.

We also studied the case of repeated games, assuming that the probability that a next round is repeated is high enough, *i*.*e*. ω = 0.9. In repeated games, the noise –understood as the probability α that individuals play the opposite action they intended to– can play an important role^52, 53^. In Fig. 4 we show the strategies that emerge in these games for different errors. One can immediately notice that there are no mixed states that emerge in repeated games with commitments. We also see that removing acceptors (RC w/o A and PC w/o A in Fig. 4) is better for the emergence of cooperation ((*P*, *C*, *D*) strategy), except when errors are high in the case of PC games as in that case one has equal probability of ending up in a pure defection or pure commitment state. In the absence of noise, repeated RC games lead to lower cooperation levels than in its one-shot counterpart. (*P*, *C*, *D*) is much stronger in PC repeated games than in PC one-shot ones since it does not need to coexist with (*A*, *C*, *D*) to avoid pure defectors; in fact, when acceptors are removed, proposers are even more successful, which is the opposite of what we showed in PC one-shot games. Note that the disadvantage of always paying $\epsilon$ in PC games is much less important in repeated games since the game is repeated for several rounds and that cost is only paid at the beginning of the commitment.

**Figure 5:**
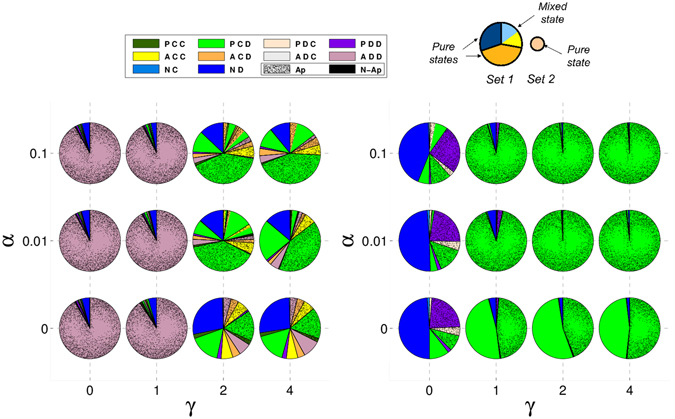
Acceptors reduce cooperation when an apology-forgiveness mechanism is introduced; defective acceptors take over the population when the apology cost is too low in RC games. Composition of the recurrent sets for RC repeated games with forgiveness. Same as Fig. 1 but for RC repeated games as a function of α and γ. Dotted (plain) sectors represent strategies that (do not) include apology. We assumed $\epsilon$ = 0.25 and *δ* = 4.

**Figure 6:**
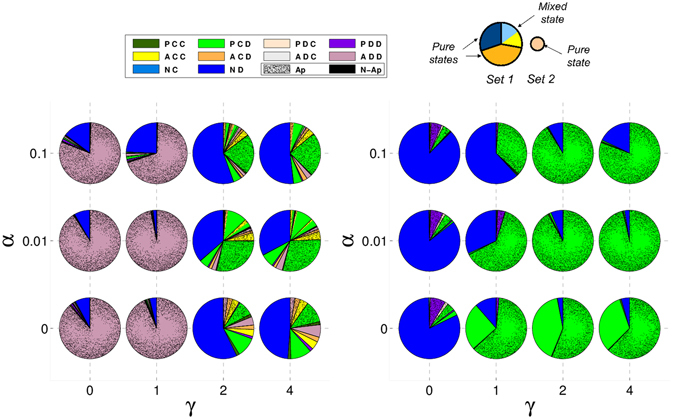
Similar to PC games, in RC games acceptors reduce cooperation in general and take over the population whenever the apology cost is too low when an apology-forgiveness mechanism is introduced. Composition of the recurrent sets for PC repeated games with forgiveness. Same as Fig. 5 but for PC repeated games.

In Figs 5 and 6 we show the results obtained for RC and PC repeated games, respectively, when the apology-forgiveness mechanism discussed in ref. 41 is introduced. We can see that apology emerges especially when noise is present.

The actual value of this noise does not make an important difference though, only its presence. Acceptors reduce the success of cooperative proposers; even when they do not obtain a high frequency in the stationary distribution, they provide advantage to pure defectors. On the other hand, as we showed before, when the apology cost is too low, *i*.*e*. γ ≲ c, individuals that try to exploit the apology-forgiveness take over the population. However, in the present study we can see that it is the acceptors who are showing this behaviour instead of the proposers.

## Discussion

We have studied the emergence of strategies in commitment games through a methodology previously developed to analyse ecosystem assembly^49^. The advantage of this methodology is the incorporation of mixed states born from the invasion of pure states.

Mixed states can have an important influence on the dynamics of the system. In the case of games where a commitment mechanism is present, mixed states highlight here the role of the acceptors –individuals who are willing to participate in commitments but only when they do not have to pay the cost to set them up. Yet their role is non-trivial as they can either promote or inhibit the emergence of cooperation.

When the commitment is defined for a one-shot game, cooperative proposers and acceptors stably coexist. This state is the most frequent or dominant among the whole set of pure and mixed states for a wide combination of parameters of the model. When acceptors are not allowed, we have shown that proposers are more successful if they only need to pay the set-up cost whenever they know that the co-player wants to commit. However, if the commitment set-up cost needs to be paid before knowing the intentions of the co-player, pure defectors take over the population in the absence of acceptors. In other words, acceptors maintain defectors away from the population and promote cooperation through their alliance with proposers in short-term agreements if the intentions of the players regarding their participation can be made known a priori, e.g. via communication or an ability to recognise the intentions of others^54^. Indeed, intention recognition plays then an important role in promoting the evolution of cooperation, as shown in previous works^22, 55^.

When commitments are made for longer relationships –repeated games–, proposers are strong enough to maintain cooperation on their own. However, acceptors make this harder. They do not coexist well with proposers and reduce the presence of the latter in the distribution, not only through their own presence in it but also indirectly making pure defectors more successful. In such long-term commitments, we also showed that an apology-forgiveness mechanism emerges when the apology cost is high enough and acceptors are not present, as in ref. 41. However, mixed states make again acceptors important actors, in this case in the regime where the apology-forgiveness is exploited: if the apology cost is too low, defecting acceptors are those that take over the population, apologising after every defection.

Deposit-refund mechanisms offer similar results as commitments do for the same set-up and compensation costs. It is also likely that these different mechanisms require different costs, since the institutions involved in their maintenance are also different.

In conclusion, the presence of mixed states can change the dynamics of commitment-based systems through the role of acceptors. In most of the scenarios, acceptors benefit from them. In other situations acceptors just undermine proposers and allow pure defectors to be successful. Commitments promote cooperation but they should be designed in such a way that they only allow acceptors to participate if the commitment is short-term and individuals are not able to know whether the others are going to participate in its establishment.

## Methods

In order to evaluate the success of different strategies under commitment games, we use a method developed in ref. 50. Just as in the stochastic finite-size population dynamics^37, 56, 57^, a graph of invasions is built, where the nodes represent the different strategies and the weighted directed edges the probability that a small fraction of individuals playing another strategy –mutants– invade the resident strategy. Yet differently from the earlier approach, invasions can now also lead to mixed states. These new mixed states are incorporated as new nodes in the graph and then the remaining strategies (pure states) will try to invade them. For every state and any invading strategy we carry out multiple realisations or runs of the process (at least 100 realisations are performed). If a run ends up in an equilibrium, a link is added to the graph going from the node corresponding to the resident population to the node corresponding to the new equilibrium. This link is weighted by the fraction of the runs that lead to it. Once the full graph is obtained, the stationary distribution of strategies is computed, including the relevant pure and mixed states. In order to compute this stationary distribution, the theory of Markov chains is applied to identify the recurrent sets (*i*.*e*. the sets of strategies that cannot be invaded by any external one), to calculate the probability of ending up in one of them, and the frequency of each strategy within each set (see ref. 50 for more details). In every realisation of the process, we assume a well-mixed finite population of *Z* individuals. Initially, 1 − *µ* of them play the resident strategy and the remaining µ the mutant one. In our analysis below, we set *Z* = 1000 and *µ* = 0.01. Individuals are repeatedly and randomly matched in pairs to play the game. After each interaction, individuals accumulate payoffs, which represent their fitness or social success. They repeat this process infinitely (*i*.*e*. until the average payoffs per round reach equilibrium) within each generation. At the end of each generation, a randomly chosen individual replaces her strategy by that of another randomly chosen individual with a probability p_*r*_ that depends on the difference of the average payoffs of such individuals Δ*W*:

4 $${p}_{r}=\frac{1}{2}(1+\frac{{\rm{\Delta }}W}{\varphi }),$$

where *ϕ* is the largest possible payoff difference. The average payoff of each individual is calculated analytically for a given composition of the population:

5 $${W}_{i}=\sum {x}_{j}{W}_{ij},$$

where *x*_*j*_ is the fraction of individuals that play strategy *j* and *W*_*ij*_ the payoff that an individual playing strategy *i* obtains against an individual playing strategy *j*.

The imitation process itself is implemented through simulations. Yet this process may become really slow when the probability of selecting two individuals that play different strategies is very small since selecting randomly pairs of individuals produces in most cases players with the same strategy. In order to solve this problem, an acceleration method is used that allows the process to quickly move beyond such situations^50^ Concretely, we calculate the probability that two individuals adopting the same strategy are selected from the population, allowing us to also determine the time that it takes until two players are selected with different strategies and jump to that point. As a result all steps leading to no change are avoided. The simulations are run until the average composition of individuals in the population remains practically constant (*i*.*e*. less than 1% of difference during 10^6^ generations), corresponding to a convergence to the equilibrium. More details can be found in the Supplementary Material.

## Electronic supplementary material

Supplementary Information
