# Supplementary material for: When agreement-accepting free-riders are a necessary evil for the evolution of cooperation
Source: Sci Rep. 2017 May 30;7:2478. doi: 10.1038/s41598-017-02625-z (PMC5449399; doi:10.1038/s41598-017-02625-z)
Supplement: Supplementary file 1 — Supplementary Information [file 41598_2017_2625_MOESM1_ESM.pdf]

Supplementary Information.
When agreement-accepting free-riders are a necessary evil for the evolution of cooperation.

Luis A. Martinez-Vaquero[α,β,*], The Anh Han[γ], Luís Moniz Pereira[λ] and Tom Lenaerts[α,β]

[α] AI lab, Computer Science Department, Vrije Universiteit Brussel, Pleinlaan 2, 1050 Brussels, Belgium

[β] MLG, Département d'Informatique, Université Libre de Bruxelles, Boulevard du Triomphe CP212, 1050 Brussels, Belgium

[γ] School of Computing, Teesside University, Borough Road, Middlesbrough, UK TS1 3BA

[λ] NOVA Laboratory for Computer Science and Informatics, Departamento de Informática, Faculdade de Ciências e Tecnologia, Universidade Nova de Lisboa, 2829-516 Caparica, Portugal

*corresponding author: l.martinez.vaquero@gmail.com

Deposit-refund mechanism

If the commitment is set up, players leave an amount δ to an institution. The institution will give back this amount if players cooperate. The only main difference from RC is that the compensation cost is not transferred from the defector to the cooperator, it is just lost [1].

The results obtained by this mechanism are very similar to those coming from RC as can be seen if Figs. S1 and S2. Note that the mixed equilibrium between (P, C, D) and (A, C, D) that can be seen in Fig. S2 corresponds to a fraction $1 - \frac{\epsilon/2}{b-c-\epsilon/2+\delta}$ of (P, C, D) .

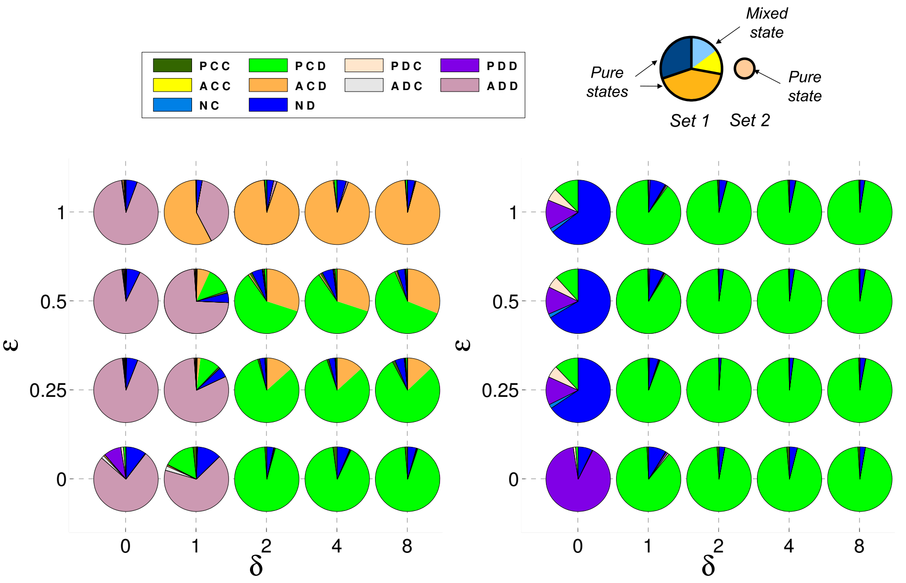

Supplementary Figure 1: The mixed state between proposers and acceptors is stable in DR games just as in the RC scenario. Composition of the recurrent sets for DR one-shot games. Same as Fig. 1 but for DR type games.

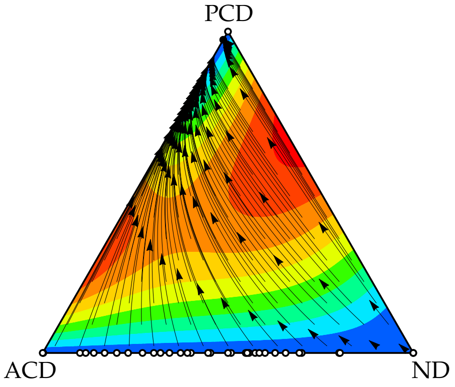

Supplementary Figure 2: Proposers maintain cooperation without the need of acceptors in DR games just as in the RC scenario. Replicator dynamics for a subset of strategies in DR one-shot games. Same as Fig. 3 but for DR games [2].

Accelerated simulations

Here we provide further details of the methodology used to carried out our simulations, which was introduced in [3]. According to Eq. 4 of the manuscript, the probability that an individual using strategy i is replaced by another with strategy $j \neq i$ if there are n_k individuals using strategy k in the population, with $\sum_j n_j = N$, is

$$\Pr(i \to j) = \frac{1}{2}\left(1 + \frac{W_j - W_i}{\phi}\right)\frac{n_i n_j}{N(N-1)}. \tag{1}$$

The probability that the composition of the population $\Delta\mathbf{n}$ changes is then

$$\Pr(\Delta\mathbf{n} \neq \mathbf{0}) = \sum_{\substack{i,j=1\\ j\neq i}}^{s} \Pr(i \to j) = \frac{1}{2N(N-1)}\left\{\sum_{\substack{i,j=1\\ j\neq i}}^{s} n_i n_j + \frac{1}{\phi}\sum_{\substack{i,j=1\\ j\neq i}}^{s}(W_j - W_i)n_i n_j\right\} = \frac{N^2 - \sum_i n_i^2}{2N(N-1)}. \tag{2}$$

where it has been taken into account that

$$\sum_{\substack{i,j=1\\ j\neq i}}^{s}(W_j - W_i)n_i n_j = \sum_{i,j=1}^{s}(W_j - W_i)n_i n_j = 0,$$

and

$$\sum_{\substack{i,j=1\\ j\neq i}}^{s} n_i n_j = \sum_{i=1}^{s} n_i(N - n_i) = N^2 - \sum_{i=1}^{s} n_i^2.$$

Combining Eqs. (1) and (2), one can deduce that the probability that i is replaced by $j \neq i$ in one time step conditioned on there being a change in the composition of the population is

$$\Pr(i \to j|\Delta\mathbf{n} \neq \mathbf{0}) = \left(1 + \frac{W_j - W_i}{\phi}\right)\frac{n_i n_j}{N^2 - \sum_k n_k^2}. \tag{3}$$

The previous process can be decompose as

$$\Pr(i \to j|\Delta\mathbf{n} \neq \mathbf{0}) = P(j|i)P(i), \tag{4}$$

where

$$P(i) = \frac{n_i\left[N - n_i + \phi^{-1}N(\overline{W} - W_i)\right]}{N^2 - \sum_k n_k^2}, \tag{5}$$

$$P(j|i) = \begin{cases} \dfrac{n_j\left[1 + \phi^{-1}(W_j - W_i)\right]}{N - n_i + \phi^{-1}N(\overline{W} - W_i)}, & \text{if } i \neq j,\\ 0 & \text{if } i = j, \end{cases} \tag{6}$$

with $\overline{W} \equiv N^{-1} \sum_k W_k n_k$. Note that $P(i)$ and $P(j|i)$ are genuine probabilities since

$$\sum_{i=1}^{s} P(i) = 1, \qquad \sum_{j=1}^{s} P(j|i) = 1, \quad i = 1, \ldots, s,$$

With this method, individuals playing strategy i are chosen with probability $P(i)$ and replaced their strategy with $j \neq i$ according to $P(j|i)$, accelerating the original process.

The time that the population remains in the same configuration can be computed as

$$t(\mathbf{n}) = \frac{\Pr(\Delta\mathbf{n} = \mathbf{0})}{\Pr(\Delta\mathbf{n} \neq \mathbf{0})} + 1 = \frac{1}{\Pr(\Delta\mathbf{n} \neq \mathbf{0})}, \tag{7}$$

and the average composition of the population $A(\mathbf{n})$ and the average time that it lasts as

$$\langle A \rangle = \tau^{-1} \sum_{\alpha=1}^{t} A(\mathbf{n}_\alpha) t(\mathbf{n}_\alpha), \qquad \tau = \sum_{\alpha=1}^{t} t(\mathbf{n}_\alpha). \tag{8}$$

The simulations are run until the average composition of individuals in the population remains practically constant (i.e. less than 1% of difference for an average time $\tau \approx 10^6$).

References

[1] Cherry, T. L. & McEvoy, D. M. Enforcing compliance with environmental agreements in the absence of strong institutions: An experimental analysis. *Environ. Resour. Econ.* **54**, 63–77 (2013).

[2] Sandholm, W. H., Dokumaci, E. & Franchetti, F. Dynamo: Diagrams for evolutionary game dynamics. *http://www. ssc. wisc. edu/˜ whs/dynamo* (2012).

[3] Martinez-Vaquero, L. A., Cuesta, J. A. & Sánchez, A. Generosity pays in the presence of direct reciprocity: A comprehensive study of 2×2 repeated games. *PLoS ONE* **7**, e35135 (2012).
